# Challenging current scientific practice: how a shift in research methodology could reduce animal use

**DOI:** 10.1038/s41684-023-01308-9

**Published:** 2024-01-03

**Authors:** S. Helene Richter

**Affiliations:** Department of Behavioural Biology, University of Münster, Münster, Germany

**Keywords:** Neuroscience, Zoology, Drug discovery, Genetics, Ethics

## Abstract

The 3R principles provide an ethical framework for animal research throughout the world. However, despite the increasing awareness of these principles, there is still a lot of room for improving their implementation, especially when it comes to reduction. By combining Bayesian statistics with a shift in experimental design, here we present an entirely new idea to reduce animal numbers within experiments.

Replace, reduce, refine: adhering to the 3R principles is nowadays key to good scientific practice in animal research all over the world. Briefly, the framework is based on the idea that if animals were to be used in scientific experiments, every effort should be made to ‘replace’ them with non-sentient alternatives, to ‘reduce’ the number of animals needed and to ‘refine’ experiments in such a way that they cause the minimum pain and distress to the experimental subjects.

## Reduction as a key principle in animal research

Despite the increasing awareness of these principles, however, there is still a lot of room for improving their implementation. This seems particularly important for the principle of reduction, because much progress has been made in both developing alternatives to animal testing (replacement) and improving animal housing and breeding conditions (refinement) over the past decade. To achieve similar goals in terms of reduction, animal researchers have repeatedly emphasized the need for a rethinking of current methodologies, mainly criticizing aspects of the planning, conduct and analysis of animal experiments (for example, see ref. ^1^). In particular, the question of how to determine the optimum sample size has been identified as being key to developing effective reduction strategies (for example, see ref. ^2^).

## The traditional way of determining sample size

Traditionally, sample size estimations follow a so-called frequentist approach, allowing to prospectively determine the smallest sample size that is sufficient to achieve a desired power (usually 80%) with an estimated effect size and a specified significance level (usually 5%; see Box 1 for definitions of key terms). This way, scientists are explicitly encouraged to carefully determine their sample sizes before the start of the experiment, preventing the use of the same ‘standard’ sample size of 8–12 animals per group, simply because of logistical, economic or political considerations. While such an approach is highly advisable to ensure an overall better planning of animal experiments, it comes with certain challenges in experimental practice. More specifically, such a priori sample size estimations force scientists to make assumptions about the expected effect size under investigation, and therefore critically rely on historical knowledge or data. This, in turn, bears the risk of observing a potential discrepancy between the prospectively assumed and the actual power. Theoretically, this discrepancy can lead to either an over- or an underestimation of the actual power, resulting in either too small or too large sample sizes, respectively (that is, using less or more animals than needed to detect a potential treatment effect).

Concerning the potential overestimation of actual power, there is indeed increasing evidence that the statistical power of animal experiments is much lower than commonly assumed a priori^2,3^. The consequences are twofold: first, many studies are dramatically underpowered in animal research, involving too few animals per group to correctly identify true treatment effects. Applying rather small sample sizes to reduce animal use might therefore have the opposite effect, as this approach undermines the scientific goal of producing sound and valid conclusions and, therefore, wastes rather than saves animals. Second, if observed effect sizes are indeed much lower than the assumed ones, this calls for a more realistic estimate of sample sizes in experimental practice, probably resulting in a drastic increase in prospectively estimated animal numbers within single experiments. However, in light of current efforts to reduce the overall animal use for research, the increase of numbers per single experiment might face resistance at least from the regulatory authorities.

Likewise, concerning the underestimation of actual power, the use of too large sample sizes also raises ethical concerns, because it is directly linked to an unnecessary waste of animals. This is particularly disquieting because a priori power calculations exclude the possibility to change animal numbers as the actual information comes in. Consequently, a priori power calculations explicitly preclude the possibility to save animals in case information changes and the discrepancy in terms of power becomes evident over the course of a running experiment. Thus, to implement a successful reduction strategy, a more flexible way of determining sample sizes that allows for reducing the number of animals within each experiment as much as possible, while at the same time guaranteeing scientific validity and reproducibility of research findings, would be desirable.

Box 1 Glossary of key terms **Bayesian updating**

The state of knowledge about quantities of interest before, or prior to a study is updated by incoming evidence to yield the state of knowledge after, or posterior to the study.

**Effect size**

A quantitative measure of the magnitude of the experimental effect.

**External validity**

The extent to which the results of an experiment provide a correct basis for generalizations to other populations and/or other environmental conditions.

**False positive rate**

The proportion of positive cases that were incorrectly identified or classified as positive in a test.

**HARKing**

Hypothesizing after the results are known.

**Interim analysis**

Analysis of data that is conducted before data collection has been completed.

**Internal validity**

Refers to whether the effects observed in a study are due to manipulation of the independent variables and not some other, unknown factors.

**P-hacking**

Any measure that a researcher applies to render a previously non-significant *P* value significant.

**Power calculation**

A statistical procedure to calculate the minimum sample size required to detect an effect of a given size.

***P*** **value**

Used in hypothesis testing to help decide whether to reject the null hypothesis; the smaller the *P* value, the more likely it is to reject the null hypothesis.

**Reproducibility**

The ability of a result to be replicated by an independent experiment in the same or different laboratory.

**Sample size**

The number of experimental units included in a study to answer the research question (often labelled with ‘*n*’).

**Significance level**

The probability of rejecting the null hypothesis when it is actually true (often labelled with ‘α’).

**Standardization**

The homogenization of the properties of any given animal (or animal population) and its environment, together with the subsequent task of keeping the properties constant or regulating them.

**Statistical power**

The probability of detecting an effect when there is actually one.

**Systematic variation**

Systematic variation of the properties of any given animal (or animal population) and its environment within a single experiment (also referred to as ‘systematic heterogenization’ in the literature).

## The use of historical data for power calculations

Lately, the increasing awareness of problems surrounding the concept of statistical significance (for example, see refs. ^4,5^) promoted the implementation of Bayesian statistical methods in experimental (animal) research (for example, see refs. ^6,7^). In particular, questionable research practices, such as ‘p-hacking’ or ‘HARKing’ (Box 1), which have their roots in frequentist inference, further triggered a discussion about the potential (mis)use of *P* values and significance thresholds. In contrast to traditional frequentist approaches, Bayesian statistics do not rely on predefined significance levels and, even more importantly, they allow experimenters to update prior probability estimates using previously collected data (for example, see refs. ^8,9^; ‘Bayesian updating’, Box 1).

In line with this reasoning, a simulation study in 2021 delineated how to best apply Bayesian priors in the context of animal research: in this study, Bayesian updating was used to include knowledge from historical experiments and thereby to limit the number of animals used in a single experiment^3^ (‘Bayesian updating’, Box 1). This way, the authors could impressively show that including historical control data in a Bayesian updating approach could halve the minimum sample size required to reach the canonical 80% power^3^. While the article received much attention upon publication, this was not the first time that the benefits of recycling historical data in the analysis of animal experiments had been demonstrated^10,11^. Previously, a report highlighted that the use of Bayesian methods can result in a more effective use of animals, either limiting the number of animals necessary to perform well-powered research or reaching higher statistical power with the same number of animals^11^. On the downside, however, such an approach critically depends on the presence of suitable data from previously performed, similar studies (see also discussion about the ‘prior-data conflict’ in ref. ^3^). In case no (suitable) historical data are available, this benefit seems to be mere theory, calling for alternative strategies to achieve a comparable reduction in animal numbers in experimental practice.

## The mini-experiment design as a tool to reduce animal numbers

In light of the widely discussed ‘reproducibility crisis’ (for example, see ref. ^12^), we recently proposed the use of so-called mini-experiment designs to introduce variation systematically and deliberately into animal experiments^13^ (for the general concept of ‘systematic variation’ or ‘systematic heterogenization’, see also refs. ^14–16^; Box 1). While this design was originally developed to counteract strict standardization regimes and increase an animal experiment’s external validity and hence its reproducibility (compare refs. ^15,16^; ‘external validity’, Box 1), it perfectly matches the above presented idea of Bayesian updating without the need for including historical data. In a mini-experiment design, a number of equivalently designed mini experiments with a new and independent set of animals are carried out consecutively over time^13^, automatically splitting an experiment into several parts and naturally allowing for interim analyses in between (‘interim analysis’, Box 1). Moreover, as the data are collected continuously over time, the experiment can be analysed after each mini experiment, incorporating prior data and thereby accumulating information step by step. Importantly, however, to avoid introducing any experimenter bias through this stepwise knowledge gain, interim analyses should ideally be conducted by a person different to the experimenter. Moreover, to further reduce the risk of scientific misconduct and improve the robustness of such a combined approach, the design of an exemplary mini experiment as well as specific stopping criteria might be predefined and formalized in preregistered protocols.

**Fig. 1:**
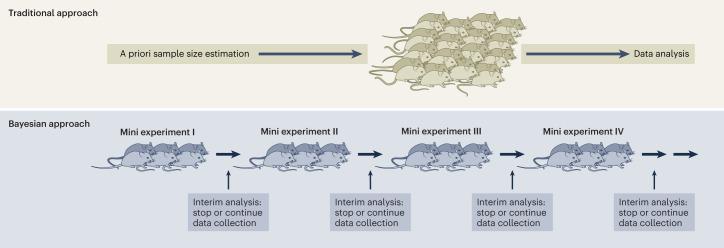
Simplified comparison of a ‘traditional approach’ and a ‘Bayesian approach’ that combines a mini-experiment design with Bayesian updating. Whereas in the traditional approach sample sizes are estimated a priori using frequentist power calculations, sample sizes can be adjusted over the course of a running experiment by conducting interim analyses in the Bayesian approach. Please note that in the latter approach, the interim analyses should ideally be conducted by a person different to the experimenter to avoid introducing any experimenter bias due to the knowledge gain over the course of the running experiment.

Following this idea, a study could then theoretically be stopped at the ‘time point of optimal information gain’, helping to identify the optimum sample size over the course of the running experiment (Fig. 1; compare with sample size re-estimation design in ref. ^17^). In terms of reduction efforts, such an approach would thus allow to adjust or even reduce animal numbers by either preventing the use of too many animals within a single experiment (due to a potential discrepancy between the prospective and the actual power) or by optimizing the knowledge gain per experiment; this would avoid the use of animals for underpowered studies and hence non-valid and non-reproducible experiments. Moreover, as this approach does not necessitate an a priori determination of sample size, it might particularly convince those researchers who traditionally tend to base their sample size decisions on habit, or on logistical or economic considerations.

Overall, the strength of this approach lies in the combination of a specific experimental design, the mini-experiment design, with Bayesian updating. Whereas the former provides a systematic way for spreading an animal experiment across time, the latter represents a more flexible way compared with frequentist methods to accumulate data and modify certain aspects of the experimental design (that is, flexible sample size adjustments and interim analyses, see below) without undermining the validity and integrity of the whole experiment. For similar reasons, the use of Bayesian methods has already been promoted in the context of human clinical trials^18^. In clinical research, it has been argued that Bayesian statistics formalizes a mathematical method for combining prior information with current information at the design stage, during the conduct of the trial and at the analysis stage, thereby allowing researchers to implement so-called adaptive trial designs (for example, see refs. ^17,18^). Furthermore, in contrast to traditional frequentist approaches, according to which multiple interim inspections bear the risk of inflating the overall false-positive rate (‘False positive rate’, Box 1), the Bayesian method has been discussed as being less affected by interim analyses^19^. Better than following a rather inflexible frequentist approach, the Bayesian approach would thus allow for analysing and stopping the experiment when the answer to a specific research question is known sufficiently well (compare with ref. ^18^).

## In a nutshell

Taken together, we here argue that the use of a mini-experiment design not only could produce better generalizable and reproducible results by systematically including variation in a single experiment, but combined with Bayesian updating, could also offer the following key advantage: by allowing the integration of prior knowledge in the analysis, a study could be stopped flexibly at some optimal point between two mini experiments, allowing experimenters to adjust or even reduce animal numbers. Although such an approach requires a rethinking of current routines and stands in contrast to what is widely done in laboratory animal practice, namely the use of a priori power calculations (in the best case) and the testing of one big batch of animals at one specific point in time, it does not necessitate the implementation of logistically expensive or complicated changes of daily routines. By spreading the experiment across time and taking new statistical paths, this approach can be implemented within each single laboratory that aims to compare, for example, the effects of two or more treatments or genotypes on a variety of different outcome measures (Fig. 1). At the downside, it might in the worst case lengthen experimental times and require the training of researchers to learn and apply Bayesian statistical methods. Overall, a reasonably feasible shift in research methodology could thus not only contribute to better reproducibility in animal research, but also pave the way for more effective reduction strategies in the best meaning of the 3R concept.
